# Supplementary material for: Genome-Wide Identification of NAC Family Genes in Oat and Functional Characterization of AsNAC109 in Abiotic Stress Tolerance
Source: Plants (Basel). 2024 Apr 3;13(7):1017. doi: 10.3390/plants13071017 (PMC11013824; doi:10.3390/plants13071017)
Supplement: Supplementary file 1 [file plants-13-01017-s001.zip › Supplemental Figures.pdf]

Figure S1. Motif analysis of *AsNAC* gene structure with its encoded protein. A. Phylogenetic tree was constructed using Neighbor-Joining (NJ) method and 1000 bootstrap tests. B. Shows the position of the 10 motifs in the protein. Different colours indicate different motifs. C. Green and yellow boxes indicate gene structures, exons and untranslated regions (UTRs).

Figure S2. Phylogenetic assessment of oat and *Arabidopsis* NAC proteins. The neighbor-joining (NJ) method (including 1000 bootstrap replications) was used to construct the phylogenetic tree. According to the phylogenetic tree, the *AsNAC* proteins were classified into 14 subgroups (indicated using different colors in the tree).

Figure S3. *AsNAC* gene duplication events. Chromosomes are indicated using different colored squares, and duplicated pairs of *NAC* genes are indicated using red lines.

Figure S4. Analysis of cis-acting elements of the *AsNAC* gene promoter. A heatmap of the number of cis elements is shown on the left. The color of the bar represents the classification, and the size of the circle represents the number of cis-acting elements. Different colored boxes indicate: blue, light-responsive cis elements; yellow, the plant growth-related cis elements; purple, stress-responsive cis element; orange, phytohormone-reactive cis elements. The histogram on the right shows a summary of each type of cis element according to the abovementioned color scheme.

Figure S5. *AsNAC* gene expression patterns under various abiotic stresses and in various tissues, as determined using RT-qPCR. *AsNAC* gene expression patterns in different tissues and under different abiotic stresses. Root (R), stem (ST), leaf (LF), spike (SP), low temperature (LT), high temperature (HT), drought (DR), drought and high temperature (DH), salt (SA), base (BA), salt and base (SB), Control (CK).
