# Supplementary figures and images for: Genome-Wide Identification of NAC Family Genes in Oat and Functional Characterization of AsNAC109 in Abiotic Stress Tolerance
Source: Plants (Basel). 2024 Apr 3;13(7):1017. doi: 10.3390/plants13071017 (PMC11013824; doi:10.3390/plants13071017)

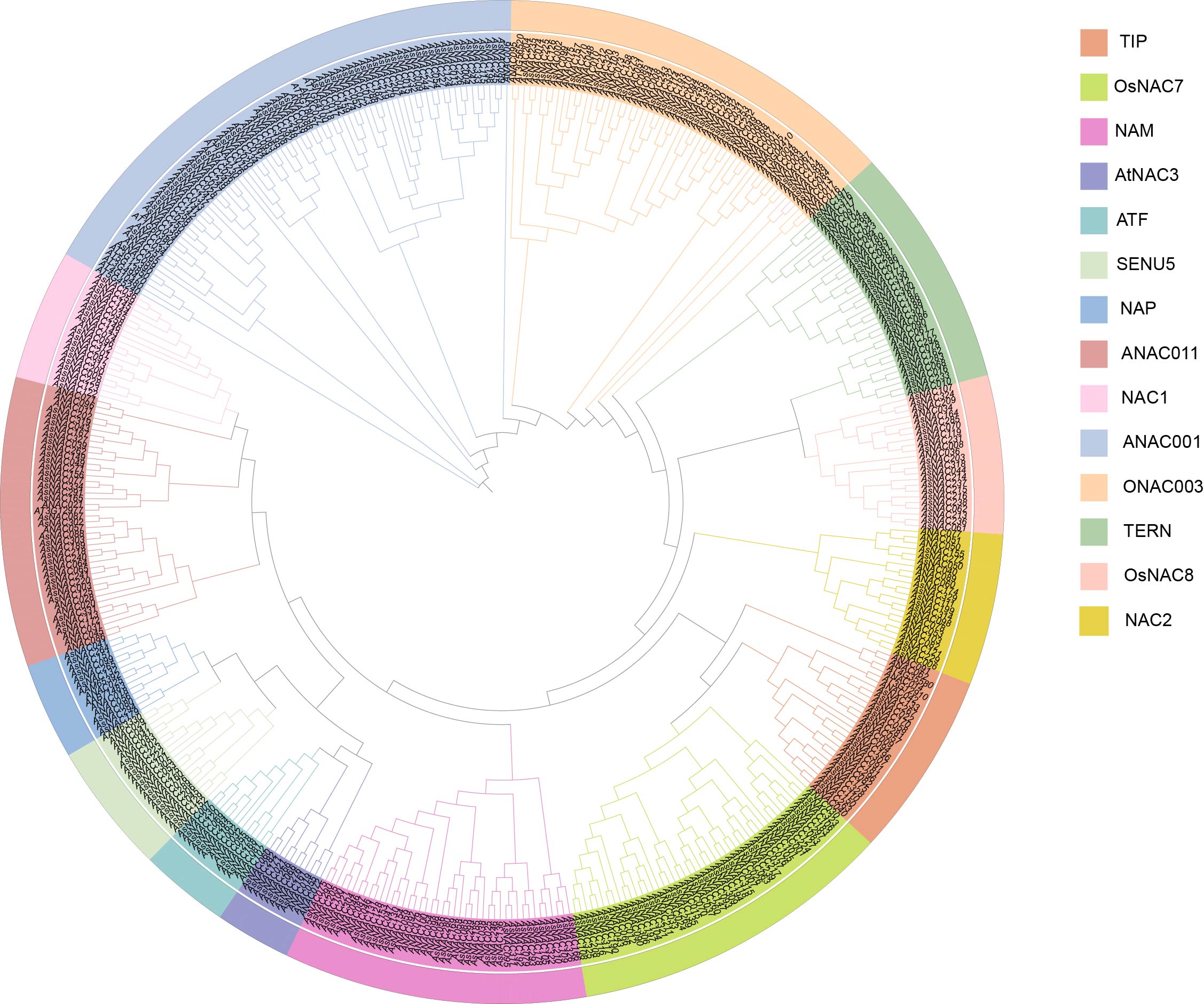

Supplement: Supplementary file 1 [file plants-13-01017-s001.zip › Figure S2.jpg]

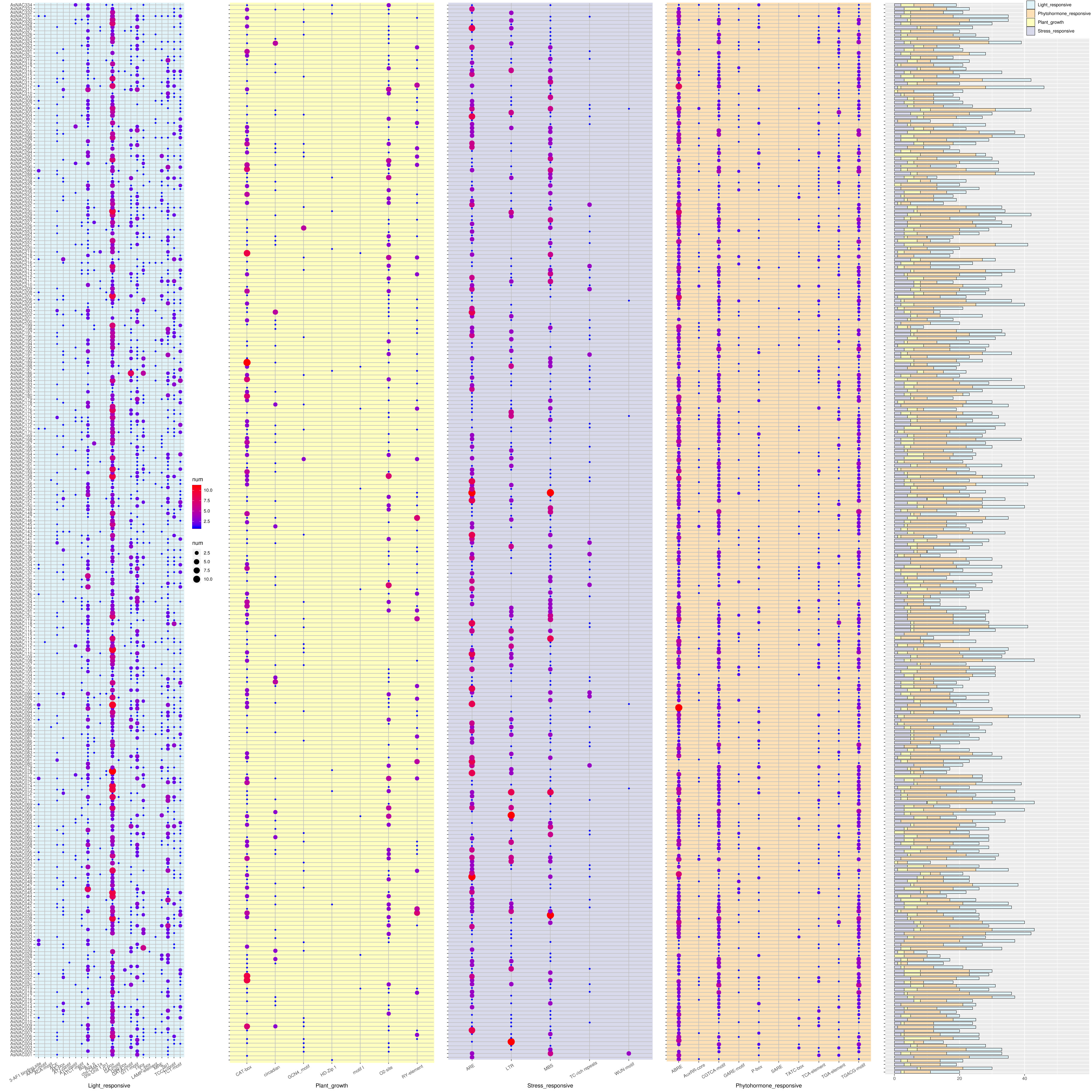

Supplement: Supplementary file 1 [file plants-13-01017-s001.zip › Figure S4.jpg]

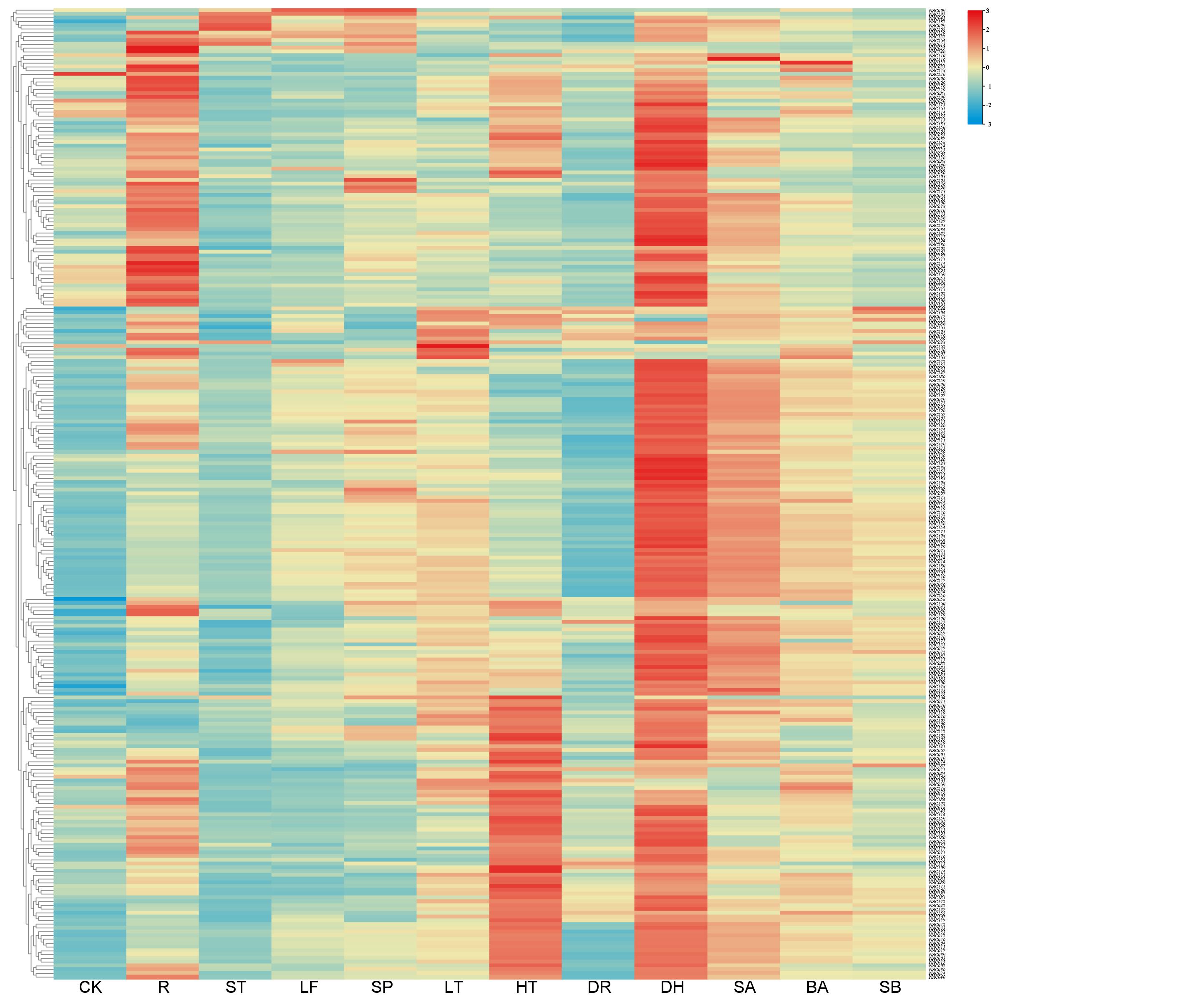

Supplement: Supplementary file 1 [file plants-13-01017-s001.zip › Figure S5.jpg]
